# The new proGAV 2.0 - a valve development based on systematical market observation

**DOI:** 10.1186/2045-8118-12-S1-P36

**Published:** 2015-09-18

**Authors:** Christoph Miethke, Thoralf Knitter

**Affiliations:** 1 Christoph Miethke GmbH & Co KG, Germany

## 

The treatment of hydrocephalus has been dramatically influenced by technical achievements which have been developed by different companies. One aspect was the principle phenomenological function of the device with the aim to lower the likelihood of negative complications like under - or overdrainage. Beneath these approaches it is important to observe the performance of the different devices based on intensive communication with the user. The manufacturer is not only obligated by law to systematically collect data about the performance of the devices. It is also important for significant improvement of the product.

Based on findings followed by systematical analyzes of revised valves, which have been send back to the manufacturer for investigation the proGAV 2.0 has been developed to address nearly all critical points, which can be recognized during the investigations. Whereas the principle hydraulic function is nearly unchanged versus the first generation proGAV the handling, the risk of blockage, the risk of damaging the valve, the way of adjusting the valve as well as the verification of the setting have been improved and the basic technical elements have been completely reworked. The first clinical experiences support the intention of the approach as well as the information gained by returned valves.

The systematical observation of the performance of medical devices offers valuable information for improvements.
